# Fractional extraction and structural characterization of opium poppy and cotton stalks hemicelluloses

**DOI:** 10.4103/0973-1296.71798

**Published:** 2010

**Authors:** Mustafa Cengiz, Ozlem Dilek Dincturk, H. Turgut Sahin

**Affiliations:** *Department of Chemistry, Faculty of Art and Sciences, S.Demirel University, East Campus, 32260 Cunur-Isparta, Turkey*; 1 *Department of Forest Products Engineering, Faculty of Forestry, S.Demirel University, East Campus, 32260 Cunur-Isparta, Turkey*

**Keywords:** Cotton stalk, fractional extraction, hemicellulose, lignocellulosic residue, opium popy

## Abstract

Hemicellulosic moieties from opium poppy and cotton stalks were solublized in water at varying alkali concentrations (NaOH) and peroxide (H_2_O_2_). The hemicelluloses were then be precipitated from the solutions by acidification. The 2.0 and 3.0% H_2_O_2_extractions resulted in a yield of 0.8 and 0.71%, respectively, accounting for 3.2 and 2.9% of the hemicelluloses present in the opium poppy stalks. A similar result was also obtained for cotton stalks. It was found that alkaline peroxide is an effective agent for solubilization of hemicelluloses from opium poppy and cotton stalks.

## INTRODUCTION

Agricultural products have been used since the dawn of civilization. Opium poppy (*Papaver somniferum* L.) has been produced in large quantities by medicinal industries. However, cotton (*Gossypium spec*.) is a primary raw material for the textile industry and has been intensively produced worldwide. The cotton fibers have advantage over synthetic fibers in the form of its high specific properties, high hydrophilic nature, high strength to weight ratio, renewable natural resource, low cost as well as easy processing.

About 9,000 dry tons of opium poppy and 2,300,000 dry tons of cotton are produced annually throughout Turkey. In general, opium poppy and cotton generates approximately 25–50% of fibrous waste residue compare to whole plant (as stalks).[1–3] However, application of agro-based residues in chemical and other process may provides alternative raw materials such as papermaking and medicinal industry, and on the other hand helps in solving some pollution problems.

Chemically, about 70–90% of opium poppy and cotton stalks are the polysacharide, much of which is in a glucose polymer. These agro-based substrates contains approximately 40–60% cellulose, another 20–40% of polysacharides are hemicelluloses; an amorphous polymer usually composed of various level of five and six carbon sugars such as xylose, arabinose, galactose, glucose, and mannose. The remainder is mostly lignin plus lesser amounts of minerals, waxes, and other compounds.[3–6]

Hemicelluloses typically located in primary and secondary cell walls are heteropolysaccharides. The chemical structure of hemicelluloses in woods are well established and contains a monomeric carbon sugars in combination with some side groups such as uronic acids, acetyl- and methyl-substituted groups. The backbone usually consists of one repeating sugar unit linked β-(1→4) with branch points (1→2), (1→3), and/or (1→6).

The principal hemicellulose in softwoods is the galactoglucomannan (approx. 20%) and its structure contains a linear chain with β-(1→4) linkages. However, the typical hardwood hemicellulose is the glucuronoxylans. This polysaccharide contains a xylan backbone of D-xylopyranose units linked β-(1→4) with acetyl groups at C–2 or C–3 of the xylose units. The detailed information on wood polysacharides can be found elsewehere.[7,8]

Although the general chemical component of annual plants and agro-based materials similar to woods, there are considerable differences in cellulose, lignin and hemicellulose contentration. It is also well known that the hemicelluloses from annual plants and agro-based materials are various and different than woods. There are a great variety of hemicelluloses with different degrees of polymerization of the monosaccharide main chain, degrees of substitution, side residues, and the side chain length. This complex and wide variety of structures has a direct effect on the hemicellulosic properties.[9]

In recent years, great interest has been taken in hemicelluloses as polymeric materials for various applications such as for production of bio oils,[10] hydrogels,[11] and bio fuels.[12] However, the hemicelluloses from opium poppy and cotton stalks are much less studied in this respect.

It has already been explained that alkaline peroxide is an effective agent for both delignification and solubilization of hemicelluloses from lignocellulosic materials.[9] Hence, the present study describes the alkaline peroxide isolation of hemicelluloses from opium poppy and cotton stalks. The experimental findings are comparatively studied by Fourier Transform Infrared (FT-IR) spectroscopy. The effect of alkaline peroxide concentration on the yield and chemical structures of the solubilized hemicelluloses are discussed. It was believed that study on hemicellulosic moieties of opium poppy and cotton stalks would make those species more desirable to manufacture value-added products with possible potential opportunities.

## MATERIALS AND METHODS

Opium poppy stalks were obtained from Gelendost-Isparta and cotton stalks were supplied from Karacaali-Adana region of Turkey. They were first dried in air and then cut into small pieces (1–3 cm). The cut samples was ground to pass a 0.8-mm size screen.

The chemicals used in this study were purchased from a chemical supply company with a purity of 95–99%, otherwise notified.

In order to study structural differences in the hemicelluloses present in the opium poppy and cotton stalks, the hemicellulosic fractions were isolated by sequential extraction. The dried powder (10 g) was first extracted with toluene–ethanol (2:1, v/v) in a Soxhlet for 6 h, and the extracted substances were allowed to dry in an oven at 60°C for 16 h. The substrates were then soaked in 300 ml distilled water at 55°C for 2 h under stirring. Water-soluble hemicelluloses were obtained by precipitation of concentrated aqueous extracts in 95% ethanol. Then, wax and water soluble free susbtrates were treated with 300 ml 0.5 M NaOH, 200 ml 0.5, 1.0, 1.5, 2.0 and 3.0% H_2_O_2_ at pH 11.5 and 2.0 M NaOH at 55°C for 2 h. After the indicated period of treatment, the insoluble residue was collected by filtration, washed with distilled water until the pH of the filtrate was neutral, and then dried at 60°C.

The hemicelluloses released were then precipitated by pouring the concentrated solution into 120 ml 95% ethanol. The precipitates that formed were recovered by filtration, washed with acidified 70% ethanol, and then air dried. Klason lignin content in hemicellulosic samples was determined according to Tappi method T 249 cm–85.

A Shimadzu IR Prestige-21 series IR spectrophotometer was used to evaluate the chemical groups on the untreated and solvent treated substrates. The data were collected in the range of 4000-400 cm^-1^wavenumber region.

## RESULTS AND DISCUSSION

The chemical constituent of the opium poppy and cotton stalk are given in Table 1. The composition (%, w/w) of the opium poppy was found to be cellulose 49.42%, lignin 25.97%, hemicelluloses 24.61%, ash 3.23%, and extractives 4.76% on a dry weight basis. However, the composition (%, w/w) of the cotton stalk was cellulose 50.21%, hemicelluloses 24.89%, lignin 24.9%, ash 2.96%, and extractives 4.53% on a dry weight basis. The results were consisted literature data, which studied earlier on similar substrates.[2,5]

**Table 1:** Chemical composition (%) of opium poppy and cotton stalks

	Opium poppy stalk	Cotton stalk
Cellulose	49.42	50.21
Lignin	25.97	24.9
Extractives (Toluen/ethanol, 2:1 by v/v)	4.76	4.53
Ash	3.23	2.96
Hemicellulose	24.61	24.89

Although extraction of hemicelluloses in lignocellulosic substrates generally involves hydrolysis of covalanet (ester) linkages to liberate them from the matrix, this is restricted by the presence of lignin in lignocellulosic matrix systems with having various lignin–hemicellulose linkages. In addition, extensive hydrogen bonds in the cell wall among polysaccharides-cell wall components may obstructed completely isolation of hemicelluloses.

Tables 2 and 3 show the yield of hemicelluloses (% dry material) in isolated hemicellulose fractions for opium poppy and cotton stalks. The sequential treatment of the extracted free substrates with distilled water, 0.5, 2.0 M NaOH and various contentartion of alkaline peroxide for opium poppy stalk at 55°C for 2 h released 3.63, 9.62, 3.7% hemicelluloses (% dry starting material), corresponding to the dissolution of 14.8, 39.1, 15.0% of the original hemicelluloses in opium poppy stalks, respectively. However, the 2.0 and 3.0% H_2_O_2_ extractions resulted in a yield of only 0.8 and 0.71% fractions, respectively, accounting for 3.2 and 2.9% of the hemicelluloses present in the opium poppy stalks. Apparently, a part of the hemicelluloses is loosely attached within the cell walls, while a major part of the hemicelluloses are embedded tightly in the cell wall. Sun and his friends (2004) speculated that it may be related to a result of a different function of polysaccharides in the cell wall.[9]

**Table 2:** The yield of hemicelluloses (% dry matter) in isolated fractions of opium poppy and cotton stalks in various solutions

	H_2_O	NaOH (0.5 M)	NaOH (2.0 M)	H_2_O_2_ Cont. (%)					Total (%)
				0.5	1.0	1.5	2.0	3.0	
Opium poppy stalk	3.63	9.62	3.7	3.02	1.12	2.01	0.8	0.71	24.61
Cotton stalk	2.8	11.81	2.2	3.38	1.21	2.1	0.8	0.59	24.89

**Table 3:** The yield of original hemicelluloses (% dry weight, w/w) solubilized during the treatment of extracted free opium poppy and cotton stalks in various solutions

	H_2_O	NaOH (0.5 M)	NaOH (2.0 M)	H_2_O_2_ Cont. (%)					Total (%)
				0.5	1.0	1.5	2.0	3.0	
Opium poppy stalk	14.8	39.1	15.0	12.3	4.5	8.2	3.2	2.9	100
Cotton stalk	11.3	47.3	8.8	13.6	4.9	8.5	3.2	2.4	100

Meanwhile, the similar treatment for cotton stalks also solubilized 2.8, 11.81, 2.2, 3.38, 1.21, 2.1, 0.8, and 0.59% hemicelluloses (% dry starting material), corresponding to release of 11.3, 47.3, 8.8, 13.6, 4.9, 8.5, 3.2, and 2.4% of the original hemicellulose in cotton stalks, respectively.

Obviously, the total yield of hemicellulosic fractions accounted for 100% of the original hemicelluloses in the cell walls of cotton and opium poppy stalks, indicating that substantial amounts of hemicelluloses were succesfully extracted sequentially with sodium hydroxide and alkaline peroxide in the increasing concentration from 0.5 to 3.0%. These results also revealed that the sequential extraction of the these materials was very effective, and the highest extraction yield was obtained with 0.5 M NaOH (39.1% for opium poppy stalk and 47.3% for cotton stalk), implying that initial extraction with 0.5 M NaOH is selective to hemicelluloses of these substrates. The similar results were also reported for sugarcane bagasse by Sun and his group (2004). It is also well known that some of the polisacharides are easily dissolved in the alkali medium degraded to form lower molecular weight products that might either remain in an insoluble form within the cell matrix or be dissolved into the liquors.[13,14]

**Figure 1:**
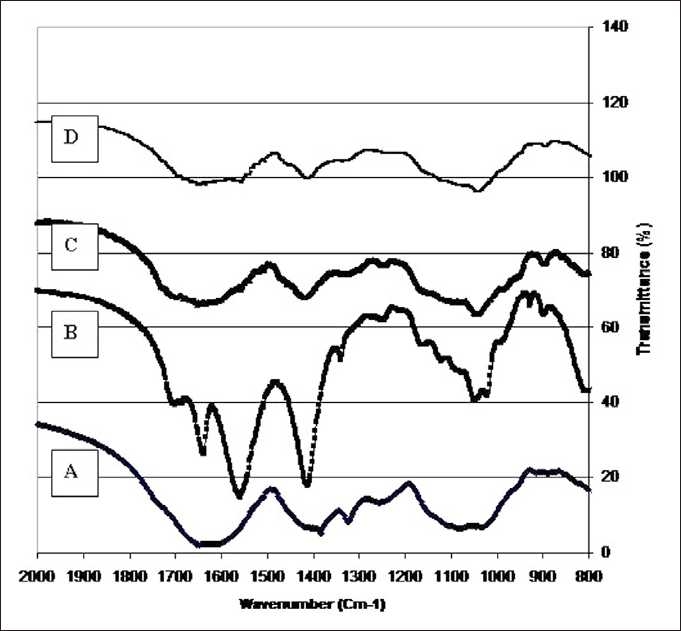
FT-IR spectra of hemicellulosic fractions released during the sequential treatment of opium poppy with water (A), 2.0 M NaOH (B), 0.5% (C) and 3.0% H_2_O_2_ (D)

Figure 1 illustrates the comparative FT-IR diagrams of hemicellulosic moieties on the fractional extracted oppium popy stalks. Those spectra clearly show the typical signal pattern for hemicellulosic moiety that are various forms of glucose, xylan and arabinose structures of the substrates which were chracterized earlier by Çopuroğlu (2004). However, the water solubilized hemicellulosic fractions shown typical stretching and bending vibrations of C–O, C–C, C–OH, and C–O–C groups at around 1000 cm^-1^ [Figure 1A]. The bands between 1175 and 1000 cm^-1^are typical of xylans. In the carbonyl stretching region, in addition to an intensive signal due to the absorbed water at 1640 cm^-1^, a small band at 1740 cm^-1^in alkali-soluble hemicellulosic fraction [Figure 1B] is assigned to the acetyl, uronic, and ferulic ester groups of the polysaccharides, whereas the absence of this signal in the spectrum of water-soluble hemicellulosic fraction demonstrated that the water treatment under the condition used cleaved this ester bond from the hemicelluloses. Figure 1 also illustrates the FT-IR spectra of hemicellulosic fractions released during the treatment with 0.5% [Figure 1C] and 3.0% H_2_O_2_ [Figure 1D]. The most obvious feature is the similarity of the spectra, indicating a similar structure of the hemicelluloses. As expected, the absence of a signal around 1720 cm^-1^for carbonyl stretching in all these two [Figure 1C and D] spectra indicated that the sequential treatments with alkaline peroxide under the conditions given did not significantly attack the glycosidic linkages and hydroxyl groups of hemicelluloses.

It is well establish by Çopuroğlu that cotton stalks contain hemicellulosic structures of arabinose, galactose, xylan, rhamnose, mannose, glucose groups with various linkages.[5] With having this informations, water and alkaline peroxide isolation of hemicelluloses from cotton stalks were conducted and the chemical structures of the solubilized hemicelluloses were comparatively studied by FT-IR.

**Figure 2:**
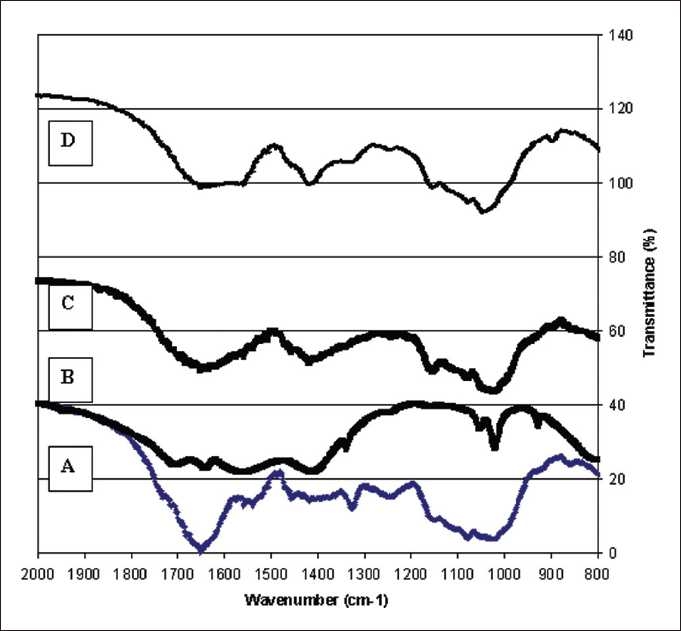
FT-IR spectra of hemicellulosic fractions released during the sequential treatment of cotton stalk with water (A), 2.0 M NaOH (B), 0.5% (C), and 3.0% H_2_O_2_ (D)

Figure 2. illustrates the comparative FT-IR diagrams of hemicellulosic fractions released during the treatment with various chemicals of cotton stalks. The water solubles hemicellulosic solutions given the absorbances at around 1474, 1394, 1268, 1169, 1096, cm^-1^in the spectra [Figure 2A]. However, the bands due to –CH_2_stretching vibrations were observed for C–H, OH, or CH_2_groups at around 1470, 1330, 1268 cm^-1^. The presence of the arabinosyl sidechains is documented by the two low-intensity shoulders at 1175 and 990 cm^-1^ [Figure 2A, C and D], The intensity changes of these two bands can be suggested to reflect the arabinosyl substituent contribution. This band gives variation in spectral shape depending on the branches at the O-2 and O-3 positions. However, a shoulder and a sharp band at 903 cm^-1^, which is should be due to the C-1 group frequency or ring frequency, is characteristic of beta-glycosidic linkages between the sugar units [Figure 2A]. The occurrence of an intensive band in Figure 2D and D shoulder in spectrum at around 1550 cm^-1^in Figure 2A is most probably due to the presence of small amounts of associated lignin in the hemicelluloses.

## CONCLUSION

It was observed that alkaline peroxide is an effective agent for solubilization of hemicelluloses from opium poppy and cotton stalks. The process can be easily applied to both substrates in regards to hemicellulose solubilization. The sequential treatment at various contentartion of alkaline peroxide released between 2.9–12.3% of the hemicelluloses in opium poppy stalks. However, the similar treatment for cotton stalks solubilized 2.4–13.6% of the hemicellulose. The alkali proxide solubilized hemicellulosic fractions for both substrates shown typical stretching and bending vibrations but some bound lignin in the isolated hemicellulosic fractions were realized by FT-IR.
